# Dewatering of *Juglans mandshurica* Wood Using Supercritical Carbon Dioxide

**DOI:** 10.3390/ma16165521

**Published:** 2023-08-08

**Authors:** Jingting Zheng, Xi Zhu, Lin Yang

**Affiliations:** 1 College of Furnishings and Industrial Design, Nanjing Forestry University, Nanjing 210037, China; jaytine99@njfu.edu.cn (J.Z.); 18173102607@163.com (X.Z.); 2 Jiangsu Co-Innovation Center of Efficient Processing and Utilization of Forest Resources, Nanjing Forestry University, Nanjing 210037, China

**Keywords:** supercritical CO_2_ dewatering, dewatering rate, moisture distribution, moisture transfer, deformation

## Abstract

Supercritical carbon dioxide (ScCO_2_), known for such features as good solubility and mass transfer properties, can be an efficient drying medium for various materials, such as wood, by filling the pore space and dissolving water in the cell cavity without altering the microstructure. In this study, two specimens of *Juglans mandshurica* wood with a length of 30 mm and 140 mm were subjected to ScCO_2_ dewatering under four different pressure and temperature conditions. The results showed that the drying rate is mainly influenced by pressure and temperature, with pressure having the more significant effect. Moreover, the efficiency of dewatering was not dependent on the sample length under the same conditions. The moisture content (MC) was the same along the longitudinal direction throughout both the surfaces and core of the wood. While there were no significant differences in dewatering rate between tangential and radial directions and lengths of samples, significant MC gradient differences were noted along wood in radial and tangential directions. During ScCO_2_ dewatering, the dominant water transfer occurred from the middle towards the end surfaces along the wood’s longitudinal directions. Furthermore, ScCO_2_ dewatering did not result in any shrinkage or significant drying stress, but it did cause some swelling in *Juglans mandshurica* wood.

## 1. Introduction

Forests cover approximately 30% of the Earth’s land, making wood the largest reserve of biomass resources found in nature. This provides a rich source of renewable and biodegradable materials for green manufacturing and environmental protection [1,2]. *Juglans mandshurica* wood, a valuable hardwood species native to eastern Asiatic regions, is notable for its unique features such as exquisite texture, dimensional stability, and ease of processing [3]. As a result, it has a wide range of applications and considerable economic value. Notably, in China, it serves as a critical component for high-end and specialized wood products such as gun stocks, models, ships, musical instruments, and cultural and sporting goods [4,5]. However, previous research has demonstrated that *Juglans mandshurica* wood poses a challenge to drying due to its special density, leading to cracking and warping when subjected to conventional drying [6].

Due to the hygroscopicity of wood’s chemical components, wood has the inherent properties of swelling and shrinkage [7,8]. Supercritical carbon dioxide (ScCO_2_) dewatering has been shown to efficiently reduce wood deformation and expedite the water removal process [9]. This dewatering technique leverages the exceptional properties of ScCO_2_, including its low supercritical temperature and pressure, non-toxicity, non-flammability, non-corrosiveness, and affordability [10,11,12]. Due to its liquid-like density and gas-like viscosity and diffusion coefficient, ScCO_2_ possesses remarkable mass transfer and solubility characteristics [13,14,15,16], for which it has been used in the chemical industry [17] and the food industry [18], making it a highly promising candidate for wood dewatering [19,20]. Although some conventional drying methods, such as high-temperature drying, radio-frequency drying, or microwave drying, may achieve a fast-drying rate, they tend to result in severe wood deformation [21,22,23,24]. In contrast, ScCO_2_ dewatering leads to lower dewatering defects and faster dewatering rates [25,26]. ScCO_2_ can remove free water from the cell cavity by cycling CO_2_ between a supercritical state and a gaseous state, thereby preserving the cell wall’s integrity and resulting in minimal dewatering defects [27,28].

Research using short samples (less than 200 mm in length) has been conducted to delve into the dewatering of wood and bamboo [21,29]. Advanced technologies like magnetic resonance imaging (MRI) and nuclear magnetic resonance (NMR) spectroscopy have also been employed to investigate the dewatering process of the sapwood of radiata pine (*Pinus radiate*) under the cyclic pressure of carbon dioxide. It was observed that CO_2_ penetrated the green sapwood through air- or water-vapor-filled cells in the latewood and then diffused into the earlywood cells adjacent to the pith side of the latewood band. The dissolved CO_2_ reduced the surface tension of water and facilitated the expulsion of sap. Upon pressure release, CO_2_ bubbles formed and expanded, and sap flowed tangentially toward the surface [30]. Utilizing fast interleaved ^1^H magnetic resonance imaging (MRI) and ^13^C nuclear magnetic resonance (NMR) spectroscopy, Robert et al. recorded the changes in cell water distribution, water flow, and CO_2_ spectra of green radiata pine wood samples during the cyclic process of supercritical CO_2_ becoming gas phase. They achieved real-time observation of supercritical CO_2_ dewatering and found that the direction and mode of water movement during wood dewatering were similar to those of traditional thermal drying by comparing ^1^H and MRI images. They also found that the final moisture content of the supercritical CO_2_ phase change dewatering process was about 40%, close to the fiber saturation point (FSP) [27]. In the existing studies, there have been some general studies on the moisture migration of wood during ScCO_2_ dewatering; however, there are limited studies on the moisture migration rules along the longitudinal direction, tangential direction, and radial direction during dewatering.

Pearson’s study concluded that pressure was the most important factor affecting dewatering efficiency but also considered factors such as temperature, pressure hold time, and pressure release time. Different wood species may have different optimal dewatering conditions [31]. Therefore, to investigate the effect of ScCO_2_ dewatering on moisture transfer in *Juglans mandshurica* wood, the samples measuring 30 mm and 140 mm in length were dewatered using supercritical CO_2_ at temperatures of 40 °C and 55 °C and pressures of 10 MPa and 20 MPa. The study examined the moisture transfer in the tangential, radial, and longitudinal directions of the wood, as well as assessing the dewatering characteristics such as drying rate, wood deformation, and drying stress.

## 2. Materials and Methods

### 2.1. Materials

**Figure 1:**
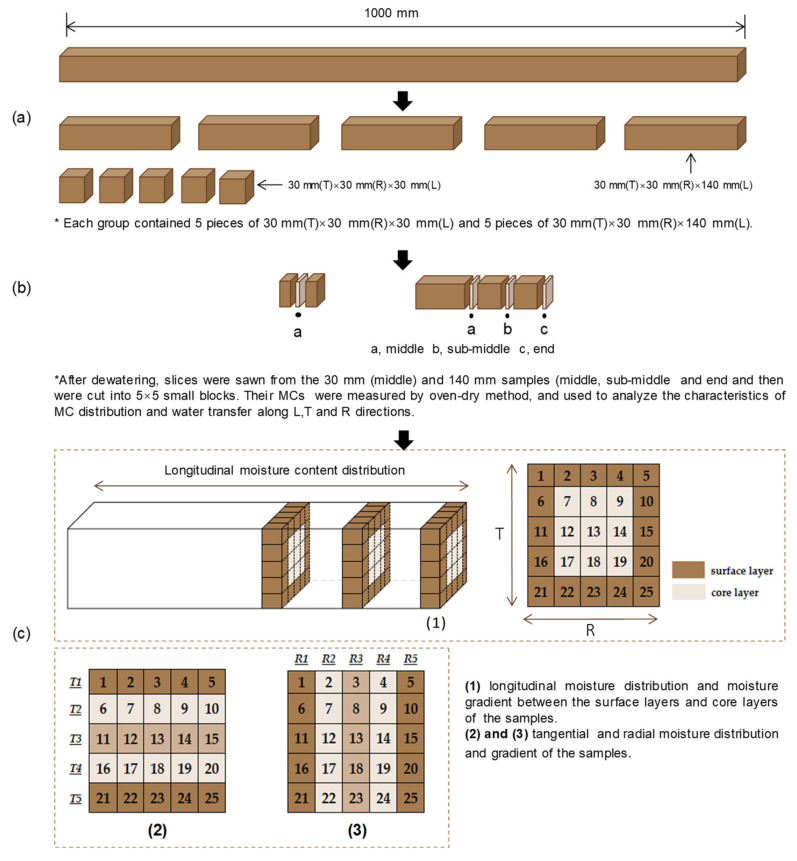
Schematic of sample preparation (**a**) sample preparation, (**b**) locations for moisture content measuring, and (**c**) moisture content distribution and gradient measuring.

*Juglans mandshurica* wood was obtained from Dandong, located in Liaoning Province, China. The collected logs were processed into sawn timbers (heartwood) having dimensions of 30 mm (tangential) × 30 mm (radial) × 1000 mm (longitudinal). Furthermore, the timbers were wrapped in plastic film and stored in a freezer maintained at 4 °C until further experimentation. Prior to the tests, five 170 mm long samples were cut from pieces of sawn timber, while two 10 mm long slices were removed from both ends of every sample. This enabled the samples to attain a final length of 140 mm. The moisture content (MC) of a sample was determined by measuring the average MC of the two slices. Each group consisted of five end-matched samples from each piece of sawn timber, and a total of four groups were prepared using four timbers in this study (Figure 1). The preparation of the 30 mm samples was similar to that of the 140 mm samples; however, all 30 mm samples were obtained from one piece of sawn timber. All samples were heartwood and free of knots and defects.

### 2.2. Equipment and Devices

**Figure 2:**
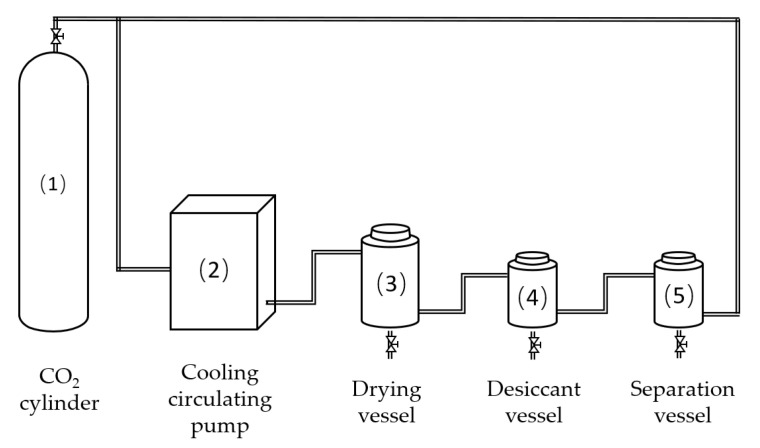
Configuration of the supercritical CO_2_ dewatering plant.

The main experimental instrument employed in this study was a supercritical CO_2_ device (HM120-50-025, Haian Hongmai Machinery Co., Ltd., Nantong, Jiangsu, China). This apparatus (Figure 2) is comprised of a CO_2_ cylinder (20 kg) (1), a cooling circulating pump (50 L/h, 50 MPa) (2), a drying vessel (2.5 L, 50 MPa, 85 °C) (3), a desiccant vessel (5 L, 50 MPa, 85 °C) (4), and a separation vessel (5). Additionally, other instruments utilized in the experiment include an electric heating oven (DHG-9643BS-III, Shanghai Xinmiao Medical Equipment Manufacturing Co., Ltd., Shanghai, China), a digital vernier caliper (CD-20CPX, Japan Mitutoyo, with an accuracy of 0.01 mm and an error of ±0.02 mm), and an electronic balance (JA5003N, Shanghai Precision Scientific Instrument Co., Ltd., Shanghai, China) with an accuracy of 0.001 g.

### 2.3. Supercritical CO_2_ Dewatering

The study comprised a total of four dewatering runs conducted at two different temperatures of 40 °C and 55 °C and two pressure levels of 10 MPa and 20 MPa. Five 30 mm end-matched samples and five 140 mm end-matched samples were selected, and their weight and dimensions were measured before they were placed into the drying vessel (3). The dimensions along the tangential and radial directions were measured at locations a, b, and c for both the 30 mm and 140 mm samples (Figure 1). The experiment was conducted according to the schedule in Table 1. Initially, liquid CO_2_ was pumped from the storage bottle (1) and gradually became a supercritical fluid, which then came into full contact with the wood inside the drying vessel (3). Once the temperature and pressure of the ScCO_2_ reached the setting value, all the samples were treated for 15 min in the drying vessel. Thereafter, the CO_2_ in the drying vessel was released gradually via a valve until its pressure reached atmospheric pressure (0.1 MPa) within a period of 10 min. Following this, all the samples were removed from the drying vessel and placed on a plastic tray for a complete CO_2_ gas emission at room temperature; the CO_2_ gas emission time was 30 min for the 30 mm samples and 60 min for the 140 mm samples. After the CO_2_ gas emission, the mass and dimensions of all the samples were measured again, and then they were cut for the determination of moisture distribution, as shown in Figure 1.

### 2.4. Moisture Content Determination in Wood

In accordance with the National Standard of GB/T1927.4-2021, the *MC* (%) of wood in this study was determined by calculating the oven-dry weight of samples using Equation (1).

*MC* = (*M*_i_ − *M*_f_)/*M*_f_ × 100% (1)

where *M*_i_ (g) is the initial mass of specimens and *M*_f_ (g) is the mass of oven-dried specimens.

To explore the MC distribution along the tangential, radial, and longitudinal directions, 10 mm thick long slices were cut from the middle portion of the 30 mm samples, as well as from the middle, sub-middle, and end portions of the 140 mm samples. These slices were subsequently numbered and divided into 25 uniformly sized blocks, following which MC was determined for each block using Equation (1). The MC distributions were then represented using these measurements, which were visually comprehensible using the software program OriginPro 2023b (OriginLab Corp., Northampton, MA, USA).

### 2.5. Deformation

Before and after dewatering, an electronic vernier caliper was used to measure the dimensions along the tangential and radial directions at locations a, b, and c for both the 30 mm and 140 mm samples (Figure 1). To determine the deformation of the samples in both tangential and radial directions, the ratio α (%) was calculated as the difference between the dimensions before and after dewatering, divided by the initial dimension using Equation (2).

*α* = (*L*_i_ − *L*_f_)/*L*_i_ × 100% (2)

where *L*_i_ is the initial tangential or radial length of samples (mm) and *L*_f_ is the final length of samples after dewatering (mm).

### 2.6. Drying Stress

After dewatering, slices with a thickness of 10 mm were sawn from the center of each 30 and 140 mm sample. The tangential dimensions of these slices were measured before proceeding to cut two strips along the tangential direction with a thickness of 2.5 mm each (from the surface to the inner part) from every slice. Then, these strips were placed at room temperature with adequate airflow for 24 h to achieve evenly distributed MC. The deflection of the strips was measured using an electronic vernier caliper; subsequently, the drying stress index (*Y*) was calculated using Equation (3).

(3) Y=f/ L×100%

where *Y* (%) is the residual stress index, *f* (mm) is the deflection of the strip after deformation, and *L* (mm) is the initial length of the strip.

### 2.7. Statistical Analysis

The statistical analysis of the treated samples was conducted using IBM SPSS Version 25 software (IBM Corporation, Armonk, NY, USA), with a 5% level of significance during the ANOVA analysis. When a statistically significant difference was observed in the one-way ANOVA, a post-hoc test was carried out to determine the specific properties that were distinct from one another.

## 3. Results and Discussion

### 3.1. Dewatering Rate and Efficiency

**Figure 3:**
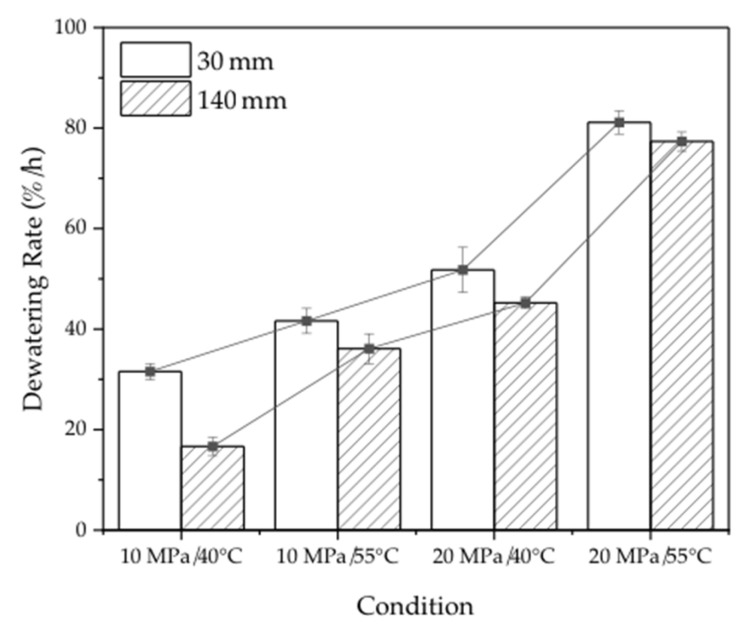
Dewatering rate of the 30 mm and 140 mm samples in four conditions.

Figure 3 and Table 2 illustrate the dewatering rates, curves, and initial and final moisture contents of the 30 mm and 140 mm samples under various dewatering conditions. The results indicate that the dewatering rate is influenced by the size of the samples, temperature, and pressure. Specifically, the short samples exhibited faster dewatering rates compared to the long samples, with the rate increasing as the temperature and pressure increased for both groups. The statistical analysis presented in Table 3 reveals that the effect of pressure on the dewatering rate is significantly greater than that of temperature. The conclusions drawn in this study are consistent with similar past studies [31]. While the dewatering rate difference between the 30 mm and 140 mm samples was 14.89%/h at 10 MPa/40 °C, this difference reduced to 5.52%, 6.57%, and 3.79% per hour at 10 MPa/55 °C, 20 MPa/40 °C, and 20 MPa/55 °C, respectively. The corresponding ratio of the dewatering rate of 30 mm to 140 mm was found to be 1.9, 1.2, 1.2, and 1.0, respectively. This trend suggests that the effect of the samples’ length on the dewatering rate decreases with increasing temperature and pressure. Additionally, it was observed that the average ratio of the dewatering rate at 20 MPa was 2.1 times that at 10 MPa, and at 55 °C, it was 1.5 times that at 40 °C. This further supports the notion that the effect of pressure is greater than the effect of temperature in the dewatering process. Increasing the temperature and pressure enhances the ability of ScCO_2_ to penetrate into the wood, allowing for more water to be expelled as the pressure is released.

**Figure 4:**
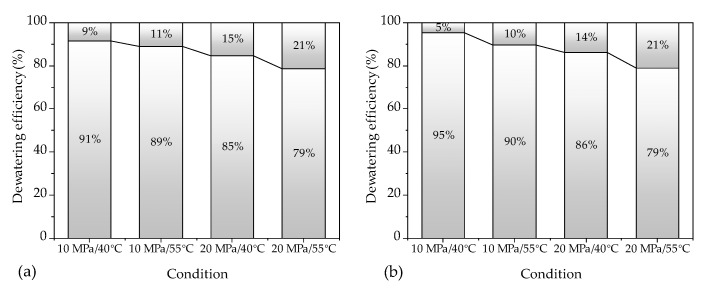
Dewatering efficiency of samples in four conditions: (**a**) 30 mm samples; (**b**) 140 mm samples.

Figure 4 displays the dewatering efficiency of all samples under four conditions. The dewatering efficiency is defined as the ratio of the dewatered water to the initial water content of the sample. The results show that the dewatering efficiency of the 30 mm and 140 mm samples was similar, except for the 10 MPa/40 °C condition. This finding indicates that the length of the sample does not significantly affect the ScCO_2_ dewatering efficiency. The result was similar to previous studies for *Eucalyptus* wood samples longer than 20 mm [12]. Furthermore, the dewatering efficiency increased with increasing temperatures and pressures of ScCO_2_.

### 3.2. Moisture Content Distribution and Gradient along Longitudinal Direction

**Figure 5:**
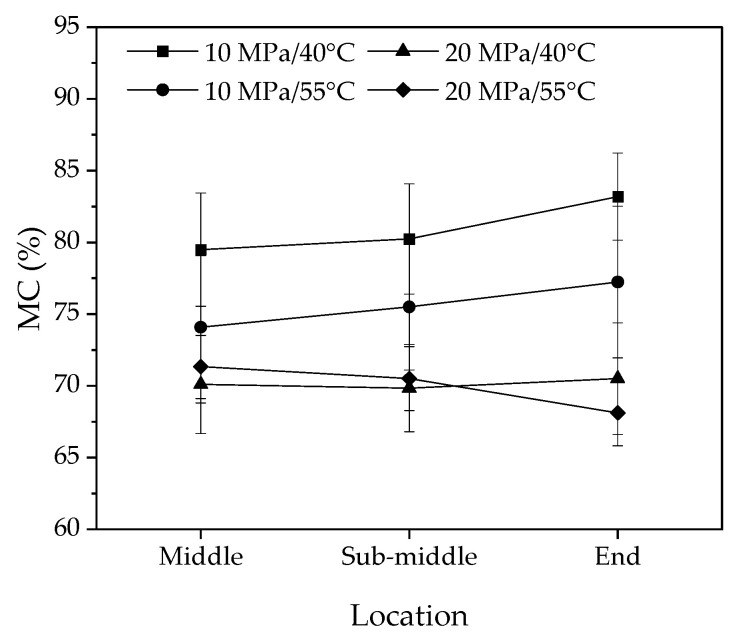
The average moisture content in the longitudinal direction for 140 mm samples after ScCO_2_ dewatering in four different conditions.

**Figure 6:**
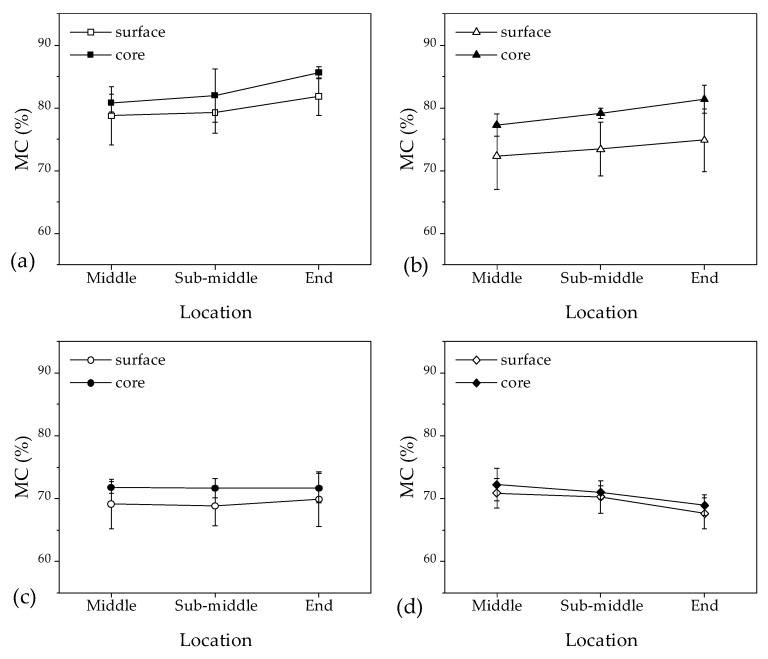
The moisture content of the surface and core layers of the 140 mm sample along the longitudinal direction is (**a**) 10 MPa/40 °C, (**b**) 10 MPa/55 °C, (**c**) 20 MPa/40 °C, and (**d**) 20 MPa/55 °C.

Figure 5 and Figure 6 show the average MC and MCs in the surface and core layers of the 140 mm samples at different positions along the longitudinal direction. Table 3 presents the analysis of variance for the effects of temperature, pressure, position in sample length, and surface to core on MCs. The results indicate that the distribution of average MCs was similar to that of MCs in the surfaces and core along the longitudinal direction. However, there were significant differences in MCs between the surface and core layers, with the largest difference observed at 10 MPa/55 °C (6%) and the smallest at 20 MPa/55 °C (2%). This suggests that the effects of temperature and pressure on MC distributions are complicated. The MC differences between the surface and core layers are more significant in Figure 6a–c than in Figure 6d. This implies that water in the surface layers of the middle, sub-middle, and end of the samples was removed faster than that in the core under the 10 MPa/40 °C, 10 MPa/55 °C, and 20 MPa/40 °C conditions. However, in the 20 MPa/55 °C condition, the water in the surface and core layers was removed at roughly the same rate.

### 3.3. Moisture Content Distribution and Gradient along Tangential and Radial Direction

**Figure 7:**
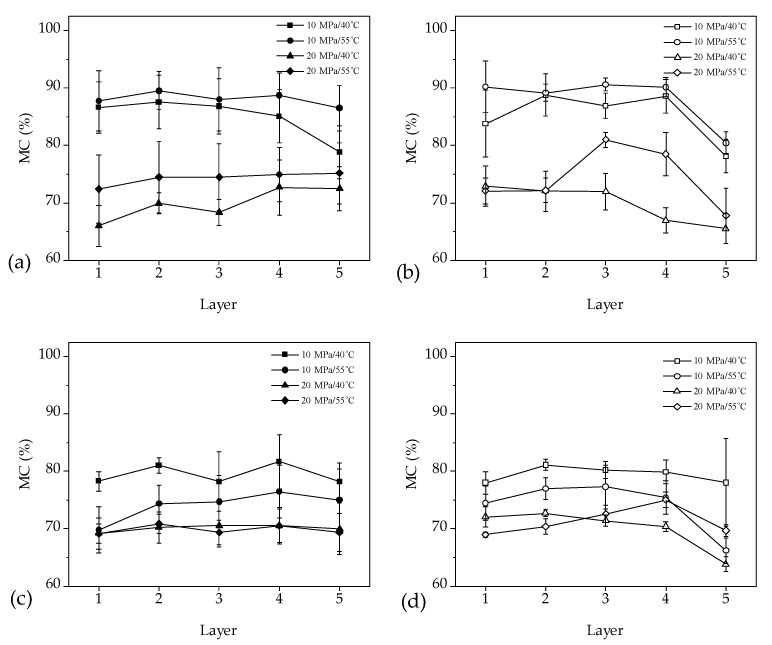
Moisture content distribution of the 30 mm samples along the (**a**) tangential and (**b**) radial directions and the 140 mm samples along the (**c**) tangential and (**d**) radial directions.

**Figure 8:**
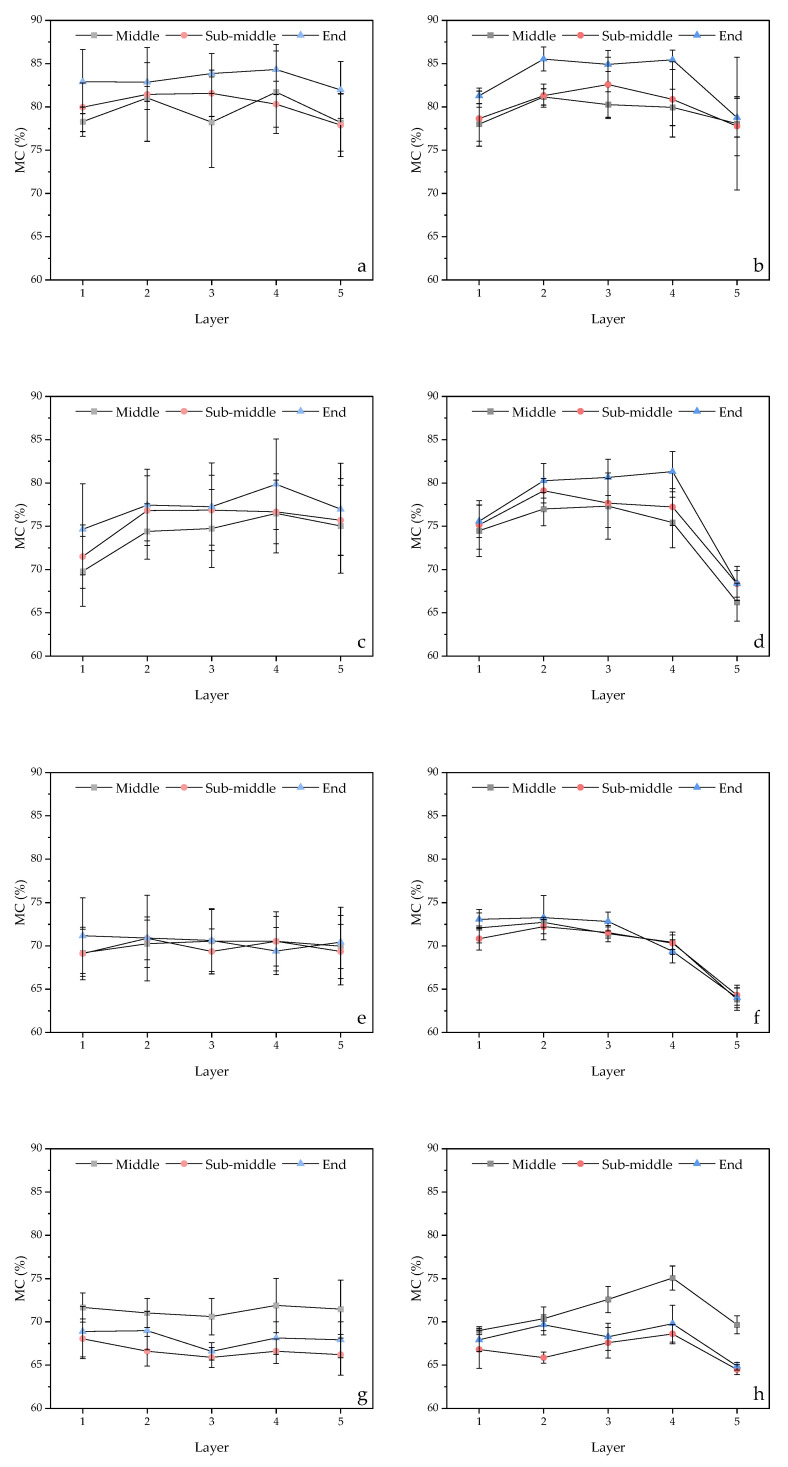
The moisture content distribution along the tangential and radial directions in the middle, sub-middle, and end sections of the 140 mm samples in four different conditions of ScCO_2_ dewatering: 10 MPa/40 °C for (**a**) radial and (**b**) tangential directions; 10 MPa/55 °C for (**c**) radial and (**d**) tangential directions; 20 MPa/40 °C for (**e**) radial and (**f**) tangential directions; and 20 MPa/55 °C for (**g**) radial and (**h**) tangential directions.

Figure 7 presents a comparison of the MC distribution in the middle of the 30 mm and 140 mm samples along the radial and tangential directions. Table 4 provides the results of the analysis of variance for the effects of temperature, pressure, direction of measurement, and layers on the MC distribution. The results indicate that the MC distribution along the tangential and radial directions in the 140 mm samples is more uniform compared to that in the 30 mm samples (Figure 7). The MC shows significant differences in different layers along the tangential and radial directions for both 30 mm and 140 mm samples (Table 4, *p* < 0.05), implying that there were substantial MC gradients in both directions, which is comparable to the MC performance in conventional kiln drying methods [32]. However, for the samples with the same length, there were no significant differences in MC distribution between the tangential and radial directions (*p* < 0.05), demonstrating that water is removed evenly along both directions. Liu et al. [12] also reported a comparable MC distribution trend in *Eucalyptus urophylla* × *E. grandis* wood during ScCO_2_ dewatering. In conventional drying methods, the moisture removal was slightly faster in the radial direction, resulting in a different distribution of MC compared to ScCO_2_ dewatering. Table 5 presents the results of the analysis of variance for the effects of temperature, pressure, direction of measurement, and layers on the MC distribution in the middle, sub-middle, and end locations of the 140 mm samples along the radial and tangential directions. The analysis shows that the sample lengths had no significant influence on the MC distribution in the transverse direction of the wood (*p* < 0.05). These findings are consistent with the results reported above, and further indicate that the water removal in the transverse direction of the wood was similar at the middle, sub-middle, and end locations, as there are no significant differences in the MC distributions along the radial and tangential directions (*p* < 0.05). Although the MCs at the end of the 140 mm samples were higher in some dewatering conditions along the radial and tangential directions, according to Figure 8, statistical analysis in Table 3 indicates that there were no significant differences in the MCs in different locations (*p* < 0.05).

Comparing the length and dewatering rate of the 30 and 140 mm samples, it was observed that the side areas of the 140 mm samples were 4.7 times greater than those of the 30 mm samples, yet the dewatering rate of the 140 mm samples was only 0.85 times lower than that of the 30 mm samples, suggesting a dominant water removal in the fiber direction with only a minor portion of water removal occurring in the transverse direction of the wood. Based on Figure 6, which shows no significant MC differences between the core and surface layers along the fiber direction of the wood, it can be inferred that water mainly transfers from the middle of the wood towards the end surfaces along the wood fiber direction.

### 3.4. Deformation

**Figure 9:**
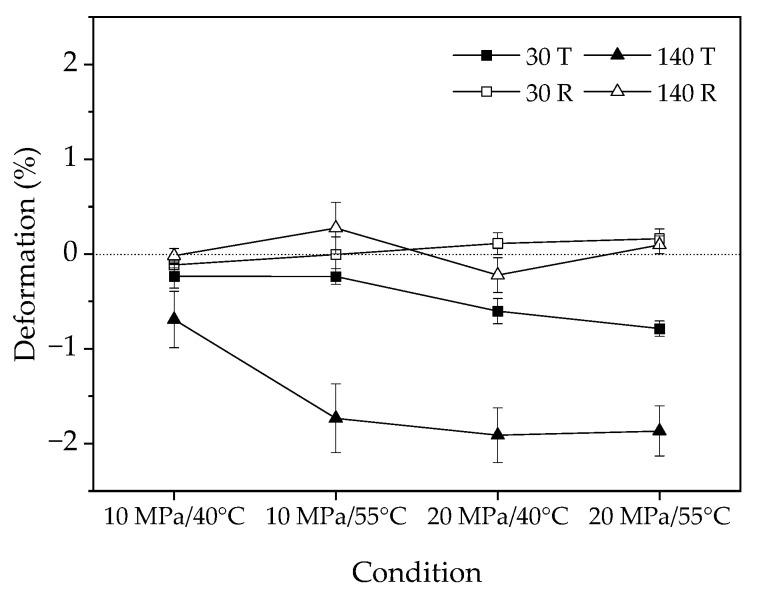
Tangential and radial deformation in four dewatering conditions.

The deformation of the 30 mm and 140 mm samples after dewatering is presented in Figure 9. Table 6 summarizes the analysis of variance for the effects of temperature, pressure, tangential or radial direction, and sample length on the deformation of the samples. In previous studies on ScCO_2_ wood dewatering, research has been conducted on wood deformation and shrinkage resulting from cell collapse, particularly in Eucalyptus nitens wood [33]. However, there has been limited investigation of the tangential and radial deformation of ScCO_2_ wood during the dewatering process. It was observed that there were minimal shrinkages in both the tangential and radial directions for samples of various sizes during the ScCO_2_ dewatering process when the moisture content (MC) of the wood was above the fiber saturation point (FSP) [12]. These findings align with previous results, except for the deformation observed in the tangential direction of the 140 mm specimens after dewatering. It is generally accepted that wood exhibits strong anisotropy during swelling and shrinkage, with the longitudinal dimension experiencing the least change, followed by the radial and then tangential directions [34]. However, in this study, the tangential direction exhibited swelling, which increased with sample length up to a maximum swelling of 2%. The tangential swelling was greater than the radial swelling for both lengths of samples. The statistical analysis in Table 6 indicates that the deformation of the samples was significantly affected only by the tangential and radial directions, suggesting that the deformation caused by ScCO_2_ differed along the transversal directions of the wood. Generally speaking, wood shrinks when its MC is below the FSP. However, no shrinkage was observed for all samples in this study as their MC was above FSP. The swelling of the samples may be attributed to the higher pressure of ScCO_2_ and the low density [35] of *Juglans mandshurica* wood.

### 3.5. Drying Stress

**Figure 10:**
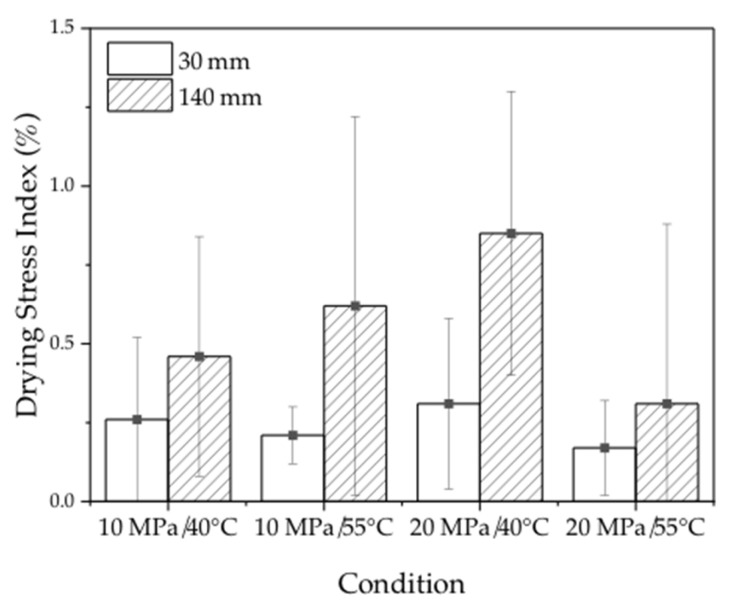
Drying stress in the surface layers of the samples.

The drying stress index in the surface layer of the 30 mm and 140 mm samples after dewatering is shown in Figure 10. Table 7 reports the statistical analysis of variance for the effects of temperature, pressure, and sample length on the drying stress of the samples. The results revealed that the drying stress indexes were small for all samples in all dewatering conditions, with a maximum value of 0.8%. Similarly, no significant difference in drying stress was observed for both sample lengths and different sizes. The conclusions from this study differ from previous findings, which suggested a consistent relationship between drying stress variations and the MC gradient trend [32]. These findings suggest that the use of ScCO_2_ as a dewatering medium induces less drying stress during the dewatering process of wood. Overall, this is a promising result, as drying stress is a major concern during the conventional kiln-drying of wood, which can lead to degradation of the wood and a reduction in its overall stability.

## 4. Conclusions

The present study investigated the dewatering characteristics of *Juglans mandshurica* wood under four different conditions, including dewatering rate, moisture distribution, deformation, and drying stress. The following conclusions can be drawn:

1. The dewatering rate of *Juglans mandshurica* wood was influenced by pressure, temperature, and sample length during the ScCO_2_ dewatering process. The dewatering rate at 20 MPa is, on average, 2.1 times higher than at 10 MPa, and at 55 °C, it is 1.5 times higher compared to 40 °C. Increasing pressure and temperature resulted in a higher dewatering rate, while longer samples decreased it.
2. The MC distributions were similar along the wood’s longitudinal direction and throughout the wood. The 140 mm samples had a more even MC distribution along the tangent and radial directions compared to the 30 mm samples, with no significant difference between the two directions for either length of sample. However, there was a significant MC gradient difference (a maximum of 6%) between the surface and core of the wood. *Juglans mandshurica* wood showed predominant moisture transfer from the middle towards the end surfaces along the wood’s longitudinal directions during ScCO_2_ dewatering.
3. ScCO_2_ dewatering induced no shrinkage but caused a certain degree of swelling in *Juglans mandshurica* wood (a maximum of 2%), likely due to the higher pressure of ScCO_2_ and the lower wood density. Moreover, the method induced less drying stress and exhibited the potential to maintain wood’s dimensional stability.
4. Dewatering wood using ScCO_2_ is a rapid and efficient method that can be further enhanced by combining it with the extraction of sap components, subsequent drying, and preservation treatment with preservatives. This not only enhances the durability of the wood but also increases its overall value.

## Figures and Tables

**Table 1:** Parameters for the ScCO_2_ dewatering process.

Process Parameter	Value
Sample length (mm)	30/140
Pressure (MPa)	10/20
Temperature (°C)	40/55
Flow (L/h)	60
Pressurization time (min)	20
Hold time (min)	15
Depressurization time (min)	10
CO_2_ emission time (min)	30/60

**Table 2:** The initial, final MC, and the dewatering rates of all samples in four conditions.

Pressure (MPa)	Temperature (°C)	30 mm			140 mm		
		Initial MC (%)	Final MC (%)	Dewatering Rate (%/h)	Initial MC (%)	Final MC (%)	Dewatering Rate (%/h)
10	40	92.36	84.48	31.55	87.83	83.67	16.66
	55	95.42	85.01	41.62	86.01	76.98	36.11
20	40	84.66	71.71	51.80	82.16	70.85	45.23
	55	94.35	74.07	81.13	91.98	72.65	77.33

**Table 3:** Variance analysis results of the 140 mm samples.

Source	Sum of Squares	DF	Mean Square	F	*p*	Significance
Factor A (Pressure)	441.956	1	441.956	92.681	0.000	*
Factor B (Temperature)	42.162	1	42.162	8.841	0.008	*
Factor C (Surface or core layer)	55.237	1	55.237	11.583	0.003	*
Factor D (The position in the length position)	5.007	2	2.504	0.525	0.600	
Error	85.835	18	4.769			
Total	134,210.449	24				

* indicates significance at 5%.

**Table 4:** Variance analysis results of the 30 mm and 140 mm samples.

Source	Sum of Squares	DF	Mean Square	F	*p*	Significance
Factor A (Pressure)	2357.292	1	2357.292	166.962	0.000	*
Factor B (Temperature)	1534.13	1	1534.13	108.659	0.000	*
Factor C (Tangential or radial direction)	0	1	0	0	1.000	
Factor D (Layer)	410.239	4	102.56	7.264	0.000	*
Factor D (The length of specimens)	27.255	1	27.255	1.93	0.167	
Error	2131.923	151	14.119			
Total	912,980.177	160				

* indicates significance at 5%.

**Table 5:** Variance analysis results of the 140 mm samples.

Source	Sum of Squares	DF	Mean Square	F	*p*	Significance
Factor A (Pressure)	2357.292	1	2357.292	358.236	0.000	*
Factor B (Temperature)	347.276	1	347.276	52.775	0.000	*
Factor C (Tangential or radial direction)	0	1	0	0	1.000	
Factor D (Layer)	256.641	4	64.16	9.75	0.000	*
Error	678.908	110	6.172			
Total	658,337.108	120				

* indicates significance at 5%.

**Table 6:** Variance analysis results for the tangential and radial deformations.

Source	Sum of Squares	DF	Mean Square	F	*p*	Significance
Factor A (Pressure)	0.323	1	0.323	2.337	0.131	
Factor B (Temperature)	0.095	1	0.095	0.69	0.409	
Factor C (Tangential or radial direction)	7.26	1	7.26	52.599	0	*
Factor D (The length of specimens)	0	1	0	0	1	
Error	10.352	75	0.138			
Total	78,070.547	80				

* indicates significance at 5%.

**Table 7:** Drying stress variance analysis results of the surface layer for the 30 and 140 mm samples.

Source	Sum of Squares	DF	Mean Square	F	*p*	Significance
Factor A (Pressure)	0.001	1	0.001	0.031	0.868	
Factor B (Temperature)	0.041	1	0.041	1.253	0.326	
Factor C (The length of specimens)	0.208	1	0.208	6.418	0.064	
Error	1.651	8				
Total	0.001	1	0.001	0.031	0.868	

## Data Availability

Not applicable.
